# Analysis of influencing factors on postoperative complications of closed approach autologous granular rib cartilage rhinoplasty and construction and verification of nomogram

**DOI:** 10.3389/fsurg.2025.1599790

**Published:** 2025-09-15

**Authors:** Ye Ju, Jinlei Wang, Xiaohong Shi, Wentao Chi, Chenguang Zhan

**Affiliations:** ^1^ Department of Otolaryngology, The 971st Hospital of Chinese People’s Liberation Army, Qingdao, China; ^2^ Department of Ward of eye, Otolaryngology and Stomatology, The 971st Hospital of Chinese People’s Liberation Army, Qingdao, China; ^3^ Department of Otolaryngology, Qingdao Municipal Hospital, Qingdao, China

**Keywords:** closed path, autologous granular rib cartilage, rhinoplasty, complication, nomogram

## Abstract

**Objective:**

To construct a nomogram prediction model based on the risk factors of complications after augmentation rhinoplasty with autogenous granular costal cartilage through closed approach, and to explore its clinical application value.

**Methods:**

From June 2022 to June 2024, 214 patients in our hospital were selected and divided into training set (*n* = 150) and verification set (*n* = 64) according to the ratio of 7:3. In the training set, the risk factors of postoperative complications were analyzed by multivariate Logistic regression, and then the nomogram prediction model was constructed. The prediction efficiency of the model is evaluated by drawing ROC curve and calibration curve, and verified in the verification set. The decision curve analysis (DCA) was used to evaluate the clinical application value of the model.

**Results:**

Complications occurred in 31 cases (20.67%) in the training group and 13 cases (20.31%) in the verification group. There was no significant difference in the incidence and clinical characteristics between the two groups (*P* > 0.05). In the training set, older age, history of chronic diseases (chronic rhinitis), long operation time, large amount of bleeding during operation and thin skin on the back of nose were independent risk factors for complications (*P* < 0.05), and a nomogram prediction model was established accordingly. The model has good calibration and fitting degree in training set and verification set (C-index index is 0.857 and 0.848, average absolute error is 0.126 and 0.090, and *χ*^2^ of Hosmer-Lemeshow test is 7.137, *P* = 0.521 and *χ*^2^ = 5.923, *P* = 0.655). The ROC curve shows that the AUC of the training set and the validation set model for predicting postoperative complications are 0.851(95% CI: 0.764–0.937) and 0.855(95% CI: 0.675–1.000), and the sensitivity and specificity are 0.880, 0.725, 0.833 and 0.692, respectively.

**Conclusion:**

The nomogram prediction model based on risk factors is helpful for early prediction of complications after augmentation rhinoplasty, providing guidance for clinical decision-making, helping to reduce the risk of complications and improving the surgical effect and patient satisfaction.

## 1 Introduction

The nose occupies a core position in facial aesthetics, and its shape has a far-reaching impact on the overall coordination of appearance. With the improvement of people's living standard and the change of aesthetic concept, the demand for rhinoplasty is increasing as an effective means to improve the shape of the nose (1). With its unique advantages, such as using its own tissue to avoid rejection, and the relatively hidden wound is conducive to recovery, the closed approach autologous granular costal cartilage augmentation rhinoplasty has been widely used in clinical practice (2). However, any operation is accompanied by certain risks, and the closed approach autologous granular costal cartilage augmentation rhinoplasty is no exception. Postoperative complications may occur, such as infection, cartilage displacement or absorption, skin redness, incision dehiscence and so on. These complications may not only make patients suffer additional physical pain and affect the recovery of nasal appearance, but also lead to psychological burden and reduce the quality of life in severe cases (3). At the same time, the occurrence of complications may also increase the medical cost and the potential risk of doctor–patient disputes. At present, the research on the postoperative complications of this operation is relatively scarce, and the related influencing factors are not completely clear. This makes it difficult for clinicians to accurately assess the surgical risk of each patient before surgery, to take effective preventive measures against potential risks during surgery, and to formulate personalized nursing plans after surgery to reduce the probability of complications (4). Therefore, it is an important problem to explore the influencing factors of postoperative complications and build a scientific and effective prediction model. As a visual forecasting tool, nomogram can integrate multiple influencing factors and predict the probability of events in an intuitive and understandable graphic way, which has been successfully applied to the risk prediction of many diseases in the medical field. It is expected to provide a convenient and efficient risk assessment method for clinicians by introducing it into the study of complications after augmentation rhinoplasty with autogenous granular costal cartilage through closed approach (5). Through the comprehensive analysis of patients' individual situation, the risk of complications can be accurately predicted, to guide doctors to formulate more reasonable surgical plans, optimize perioperative management, realize personalized medical care, minimize the risk of complications, improve the success rate of surgery, and protect patients' physical and mental health.

## 2 Materials and methods

### 2.1 Study population

Retrospective collection of clinical data from 214 patients who underwent closed approach autologous rib cartilage rhinoplasty in our plastic and cosmetic surgery department from June 2022 to June 2024. Inclusion criteria: 1. age between 18 and 50 years old, 2. first time receiving closed approach autologous granular rib cartilage rhinoplasty, 3. complete clinical data. Exclusion criteria: 1. concurrent severe dysfunction of important organs such as heart, liver, and kidney, 2. suffering from hematological disorders or abnormal coagulation function, 3. there are acute and chronic infectious diseases in the nose, 4. having mental illness or cognitive impairment, unable to cooperate with postoperative follow-up. The patients were divided into a training set (*n* = 150) and a validation set (*n* = 64) at a ratio of 7:3 using the random number table method. This study was approved by the hospital's ethics committee, and all patients signed informed consent forms.

### 2.2 Surgical methods

All surgeries are performed by the same experienced team of plastic surgeons. The patient is placed in a supine position, and after successful general anesthesia, a 2–3 cm incision is made on the surface of the 6th and 7th rib cartilage on the right side. The skin, subcutaneous tissue, and rib cartilage membrane are sequentially cut open, and an appropriate amount of rib cartilage is obtained by blunt dissection. Carefully trim the cut rib cartilage into particles of appropriate size and place them in physiological saline solution for later use. Make an incision at the base of the nasal columella or the inner edge of the nostril, carefully separate the fascial cavity of the nasal dorsum, evenly fill the prepared autologous granular rib cartilage in the nasal dorsum and nasal tip areas, adjust the shape to satisfaction, and then suture the incision layer by layer. Postoperative routine placement of drainage strips and pressure bandaging. During the dissection process, special attention is paid to avoiding damage to the intercostal nerves and the nerve branches innervating the serratus anterior muscle. The intramuscular nerve distribution of the serratus anterior muscle, as detailed by Yi et al. (6), provides critical anatomical guidance for this procedure—accurate knowledge of these neural pathways helps minimize iatrogenic nerve injury, thereby reducing the risk of donor-site complications such as chronic chest wall pain.

### 2.3 Definition of postoperative complications

Postoperative complications in closed approach autologous granular rib cartilage rhinoplasty were categorized and defined as follows: 1. early postoperative complications: infection (cellulitis or abscess formation confirmed clinically), hematoma/seroma (localized blood/fluid collection requiring intervention), skin redness/swelling (persistent inflammation >2 weeks), incision dehiscence (wound separation needing resuturing) and prolonged pain/hypersensitivity (>1 month). 2. Graft-related complications: cartilage resorption [quantified by 3D volumetric analysis as mild (<20%), moderate (20%–50%), or severe (>50%)], displacement/malposition (radiographic/clinical evidence of graft shifting), visibility/irregularities (palpable/visible contour issues), overcorrection/undercorrection (aesthetic imbalance) and fracture/fragmentation. 3. Donor-site complications: chest wall deformity, chronic pain, and pneumothorax. 4. Functional/aesthetic concerns: nasal obstruction, asymmetry, skin necrosis, and hypertrophic scarring. 5. Systemic complications: deep vein thrombosis (DVT), pulmonary embolism (PE) and anaphylaxis. Patients who experienced any of the above complications were included in the complication group. Otherwise, they were classified as the no-complication group.

### 2.4 Data collection

Collect detailed general information of patients, including age, gender, height, weight, smoking history, alcohol consumption history, chronic medical history (chronic rhinitis/rhinitis); Surgical related information, such as surgery time, intraoperative blood loss, cartilage granule filling amount, rib cartilage extraction amount; Preoperative nasal basic conditions (including nasal dorsal skin thickness, nasal facial angle, nasal frontal angle, nasal labial angle, nasal columella upper lip angle, nasal tip protrusion); Postoperative nursing compliance is classified into good compliance and poor compliance based on a comprehensive evaluation of the patient's nursing cooperation during the postoperative follow-up process. All patients were followed up regularly after surgery, with a total follow-up period of 6 months. The follow-up schedule was as follows: once a week within 1 month after surgery, once every 2 weeks from 1 to 3 months after surgery, and once a month from 3 to 6 months after surgery. During the follow-up period, all complications were recorded, including early complications (occurring within 1 month after surgery) and late complications (occurring from 1 month to 6 months after surgery, such as delayed cartilage resorption, and displacement).

### 2.5 Statistical analysis

Data were analyzed using SPSS 26.0 and R 4.2.3. Continuous variables were reported as mean ± standard deviation (SD) (if normally distributed, assessed by Shapiro–Wilk test) or median (interquartile range, Q1, Q3) (for non-normally distributed data), with between-group comparisons using independent *t*-tests or Mann–Whitney *U* tests, respectively. Categorical variables were compared via chi-square tests or Fisher's exact tests, and count data were expressed as the number of cases (percentage). Multivariate Logistic regression analysis was used to screen the risk factors of complications, and the difference was statistically significant (*P* < 0.05), and variance inflation factors (VIF) were calculated to exclude multicollinearity (VIF threshold <5). The final predictors were incorporated into a nomogram developed using the R “rms” package. Model performance was rigorously evaluated through multiple approaches: discrimination was assessed via the area under the receiver operating characteristic (ROC) curve (AUC) with 95% confidence intervals derived from 1,000 bootstrap samples, along with sensitivity, specificity, and predictive values; calibration was examined using bootstrap-corrected calibration curves, Brier scores, Hosmer-Lemeshow goodness-of-fit test (*P* > 0.05 indicating good calibration), and calibration slope analysis; potential overfitting was addressed through optimism-adjusted performance metrics using bootstrap internal validation; and clinical utility was determined via decision curve analysis (DCA). All statistical tests were two-sided, with a significance level set at *α* = 0.05.

## 3 Results

### 3.1 Baseline characteristics in training set and validation set

A total of 214 patients were included in the study. There were 31 cases (20.67%) of complications in the training set and 13 cases (20.31%) in the validation set. There was no statistically significant difference in the incidence of complications and clinical characteristics between the training set and the validation set (all *P* > 0.05) (Table 1).

**Table 1:** Comparison of clinical features between training set and validation set.

Index		Training set (*n* = 150)	Validation set (*n* = 64)	*t*/*χ*²	*P*
Age (years)		29.32 ± 5.11	28.89 ± 4.87	0.571	0.568
Height (cm)		165.35 ± 7.23	164.52 ± 6.87	0.780	0.436
Weight (kg)		58.67 ± 8.36	57.95 ± 7.88	0.586	0.558
Gender	Male	78 (52.00)	33 (51.56)	0.003	0.953
	Female	72 (48.00)	31 (48.44)		
Chronic diseases history	Rhinitis	12 (8.00)	5 (7.81)	0.002	0.962
	Allergic rhinitis	9 (6.00)	4 (6.25)	0.058	0.808
Smoking history	Yes	22 (14.67)	9 (14.06)	0.013	0.908
	No	128 (85.33)	55 (85.94)		
History of drinking	Yes	25 (16.67)	10 (15.63)	0.035	0.850
	No	125 (83.33)	54 (84.38)		
Surgical duration (min)		105.45 ± 12.34	104.89 ± 11.79	0.308	0.758
Intraoperative bleeding volume (ml)		32.54 ± 8.44	33.08 ± 7.98	0.435	0.663
Thickness of nasal dorsal skin (mm)		2.12 ± 0.35	2.09 ± 0.32	0.588	0.556
Removal amount of rib cartilage (g)		3.56 ± 0.66	3.51 ± 0.64	0.512	0.609
Cartilage granule filling amount (ml)		4.21 ± 0.88	4.18 ± 0.85	0.230	0.817
Nasal angle (°)		133.25 ± 0.85	133.12 ± 0.91	1.002	0.317
Rhino frontal angle (°)		29.37 ± 6.07	30.05 ± 5.79	0.760	0.447
Nasal and Lip Angle (°)		102.85 ± 5.06	103.41 ± 5.27	0.732	0.464
Upper lip angle of nasal columella (°)		88.47 ± 10.36	90.32 ± 8.87	1.246	0.214
Nose tip protrusion (°)		0.52 ± 0.07	0.51 ± 0.08	0.916	0.360
Postoperative nursing compliance	Poor	30 (20.00)	13 (20.31)	0.002	0.958
	Good	120 (80.00)	51(79.69)		

### 3.2 Univariate analysis in the training set

In the training set, the results of univariate analysis showed that age, chronic medical history (chronic rhinitis), chronic medical history (allergic rhinitis), surgery time, intraoperative blood volume, thickness of nasal dorsal skin, cartilage granule filling amount and postoperative nursing compliance were associated with the occurrence of complications (all *P* < 0.05) (Table 2).

**Table 2:** Univariate analysis of postoperative complications in the training set.

Indicators		Complications group (*n* = 31)	Non complication group (*n* = 119)	*t*/χ²	*P*
Age (years)		31.51 ± 6.22	28.78 ± 4.89	2.610	0.010
Height (cm)		165.13 ± 7.06	164.79 ± 7.31	0.232	0.816
Weight (kg)		58.34 ± 8.22	58.77 ± 8.42	0.254	0.799
Gender	Male	17 (54.84)	61 (51.26)	0.126	0.722
	Female	14 (45.16)	58 (48.74)		
Chronic diseases history	Rhinitis	7 (22.58)	5 (4.20)	8.928	0.002
	Allergic rhinitis	5 (16.13)	4 (3.36)	5.024	0.025
smoking history	Yes	5 (16.13)	17 (14.29)	0.001	0.978
	No	26 (83.87)	102 (85.71)		
History of Drinking	Yes	6 (19.35)	19 (15.97)	0.203	0.652
	No	25 (80.65)	100 (84.03)		
Surgical duration (min)		112.44 ± 15.34	103.87 ± 11.06	3.526	0.001
Intraoperative bleeding volume (ml)		37.22 ± 10.02	32.65 ± 7.99	2.684	0.008
Thickness of nasal dorsal skin (mm)		2.09 ± 0.33	2.31 ± 0.42	2.704	0.007
Removal amount of rib cartilage (g)		3.58 ± 0.65	3.55 ± 0.66	0.226	0.821
Cartilage granule filling amount (ml)		4.45 ± 0.95	4.02 ± 0.84	2.469	0.014
Nasal angle (°)		133.27 ± 0.88	133.24 ± 0.85	0.173	0.862
Rhinofrontal angle (°)		29.55 ± 6.12	29.37 ± 6.07	0.146	0.883
Nasal and Lip Angle (°)		103.10 ± 5.15	102.79 ± 5.09	0.301	0.763
Upper lip angle of nasal columella (°)		89.13 ± 10.05	88.32 ± 10.44	0.387	0.698
Nose tip protrusion (°)		0.53 ± 0.07	0.52 ± 0.07	0.708	0.479
Postoperative nursing compliance	Poor	11 (35.48)	19 (15.97)	5.855	0.015
	Good	20 (64.52)	100(84.03)		

### 3.3 Multivariate logistic regression analysis in the training set

Multivariate logistic regression analysis was conducted with the occurrence of complications as the dependent variable, and factors with *P* < 0.05 in univariate analysis as independent variable. The results showed that age, chronic medical history (chronic rhinitis), long surgical time, excessive intraoperative bleeding, thin skin thickness on the nasal back, high cartilage granule filling volume and poor postoperative nursing compliance were independent risk factors for complications in patients undergoing closed approach autologous rib cartilage rhinoplasty (all *P* < 0.05) (Table 3). The tolerance of each variable in the regression model was >0.1, VIF was <10, conditional index was <30, and the variance ratio of multiple covariates under the same eigenvalue was >50%. Therefore, there was no collinearity among the covariates.

**Table 3:** Multivariate logistic analysis of postoperative complications in the training set.

Indicators	*β*	S.E	Wald	*P*	OR	95%CI
Age	0.110	0.049	5.100	0.024	1.116	1.015–1.228
Chronic medical history (chronic rhinitis)	3.421	1.525	5.036	0.025	30.608	1.542–607.493
Surgical time	0.049	0.021	5.470	0.019	1.051	1.008–1.095
Intraoperative bleeding volume	0.088	0.030	8.437	0.004	1.092	1.029–1.158
Thickness of nasal dorsal skin	−1.561	0.659	5.612	0.018	0.210	0.058–0.764

### 3.4 Construction of nomogram prediction model for postoperative complications

**Figure 1:**
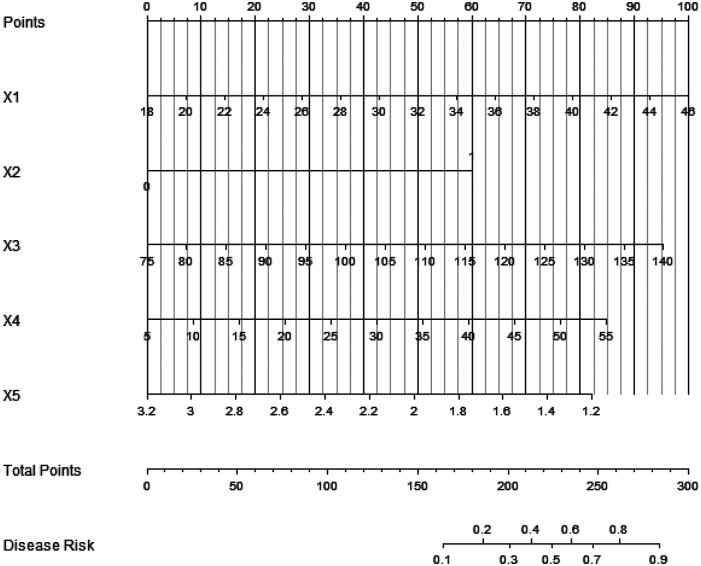
Nomogram prediction model of postoperative complications [X1: Age, X2: chronic medical history (chronic rhinitis), X3: surgical time, X4: intraoperative bleeding volume, X5: thickness of nasal dorsal skin].

Based on the independent risk factors identified by multivariate logistic regression analysis, a nomogram prediction model of complications after closed approach autogenous costal cartilage augmentation rhinoplasty was constructed, and the independent risk factors in the model were assigned, and the total score of predicting complications was calculated, which was reflected by the probability of predicting complications. A higher total score indicates a greater predicted risk of postoperative complications (Figure 1).

### 3.5 Evaluation and validation of nomogram prediction model

**Figure 2:**
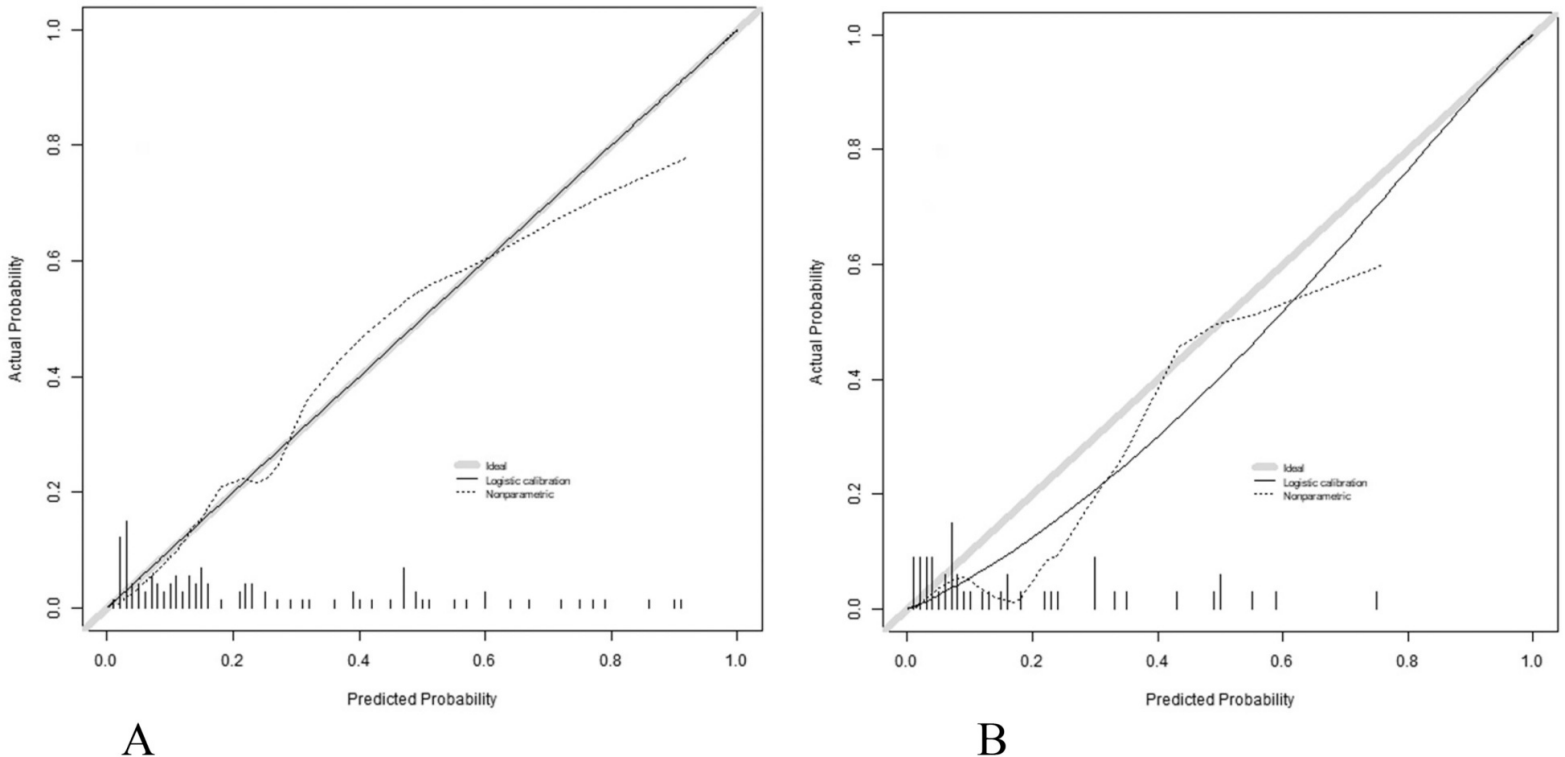
Calibration curve in training set **(A)** and validation set **(B)**.

**Figure 3:**
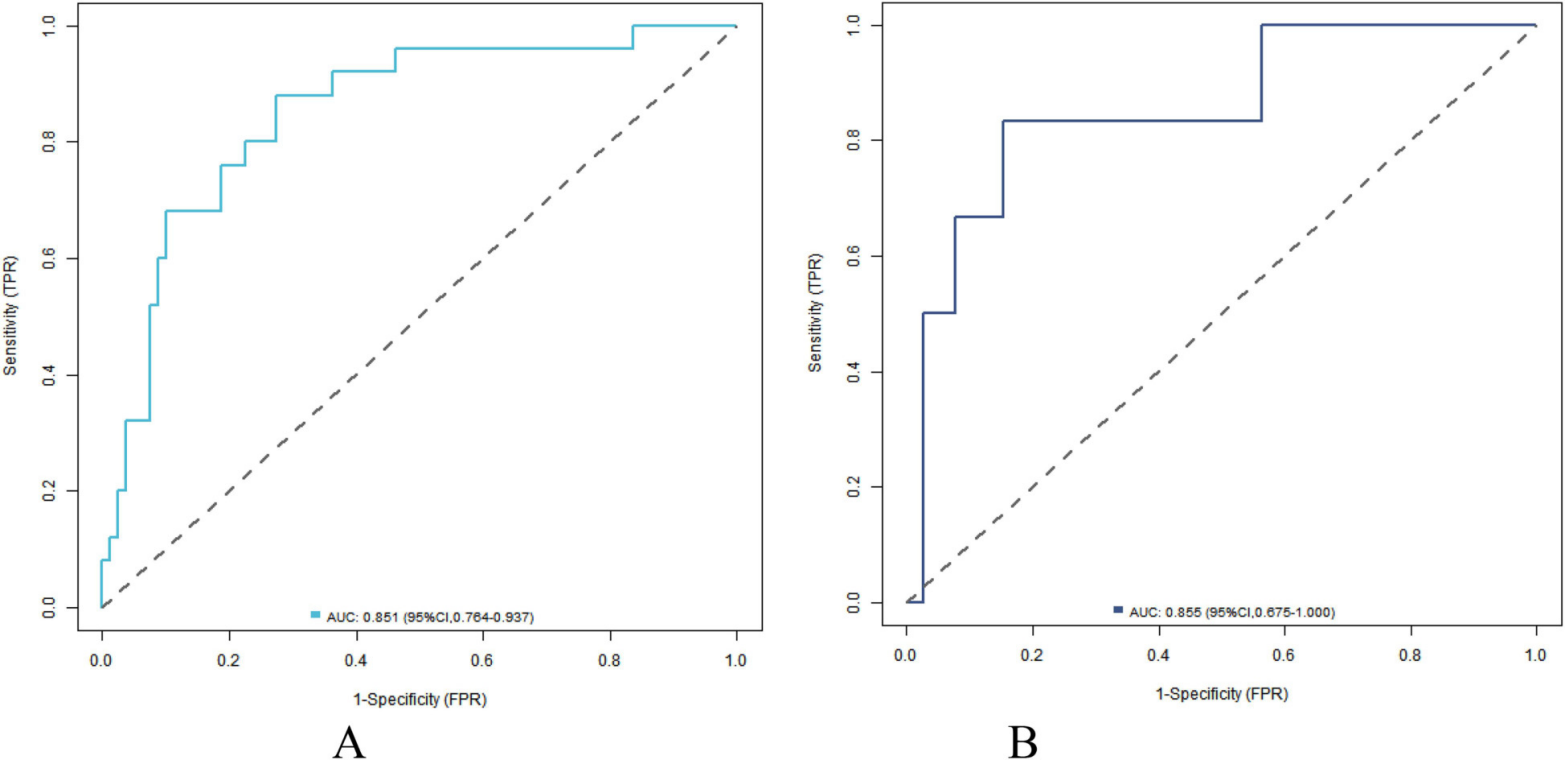
ROC curve in training set **(A)** and validation set **(B)**.

Nomogram model has good calibration and fitting degree in training set and validation set (C-index index is 0.857 and 0.848 respectively, the average absolute error of fitting degree between predicted value and real value is 0.126 and 0.090 respectively, and the results of Hosmer-Lemeshow test are *χ*^2^ = 7.137, *P* = 0.521 and *χ*^2^ = 5.923, *P* = 0.655 respectively (Figure 2). ROC curve shows that the AUC of Nomogram model in training set and validation set for predicting the complications after closed approach autogenous costal cartilage augmentation rhinoplasty is 0.851(95% CI: 0.764–0.937) and 0.855(95% CI: 0.675–1.000), respectively, and the sensitivity and specificity are 0.880, 0.725 and 0.833 (Figure 3).

### 3.6 Decision curve analysis of nomogram line prediction model

**Figure 4:**
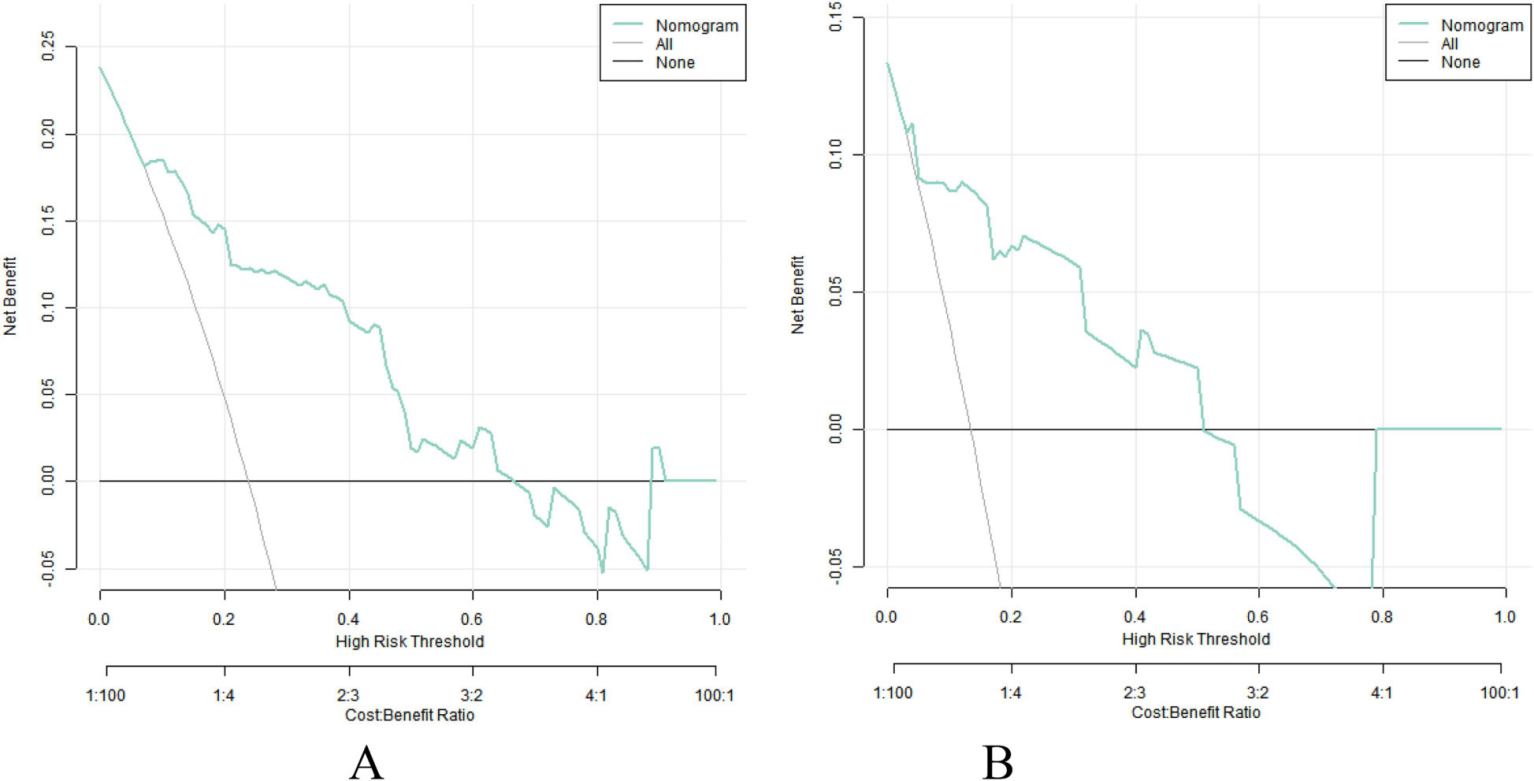
Decision curve in training set **(A)** and validation set **(B)**.

The decision curve analysis shows that when the threshold probability is about 0.10–0.90, the nomogram model constructed in this study has more clinical benefits than the decision that all patients have complications or all patients have no complications before operation (Figure 4).

## 4 Discussion

In this study, the risk factors of complications after closed approach autologous granular costal cartilage augmentation rhinoplasty were deeply analyzed, and a nomogram prediction model was successfully established. Through the research, it is found that older age, history of chronic diseases (chronic rhinitis, allergic rhinitis), long operation time, large amount of bleeding during operation, thin skin on the back of nose, large amount of cartilage particles and poor postoperative nursing compliance are independent risk factors for complications of patients undergoing this operation.

Age is one of the important factors affecting postoperative complications. With the increase of age, the physiological functions of human body gradually decline, the function of immune system declines and the ability of tissue repair weakens. In rhinoplasty, the wound healing speed of older patients is relatively slow and their anti-infection ability is reduced, which makes the risk of complications such as infection increase significantly (7). At the same time, the skin elasticity will deteriorate with age, which makes it difficult to fix and support the filled cartilage particles, and increases the possibility of cartilage displacement and absorption (8). Having a history of chronic diseases, especially chronic rhinitis, has a noticeable influence on the occurrence of complications after rhinoplasty. Chronic rhinitis will lead to chronic inflammation of nasal mucosa, impaired barrier function of mucosa and decreased resistance. When performing rhinoplasty, the surgical area relates to the nasal cavity, and inflammation is easy to spread to the surgical site, causing complications such as infection (9). In addition, frequent actions such as blowing your nose and sneezing in patients with rhinitis may affect the stability of the nose after operation, interfere with the normal healing of wounds, and further increase the incidence of complications (10). Long operation time is another key risk factor for postoperative complications. The prolonged operation time means that the operation will cause more trauma to the tissue and the operation area will be exposed to the air for a longer time, which greatly increases the chance of bacterial infection. Long-term operation may also damage the blood supply of local tissues and affect the repair and healing process of postoperative tissues. For example, during a long period of surgery, local tissues may be disordered in cell metabolism due to ischemia and hypoxia, which will affect the survival and growth of cartilage, thus causing complications such as cartilage absorption and displacement (11). The amount of intraoperative bleeding is also closely related to the occurrence of postoperative complications. Massive bleeding during operation may lead to the formation of local hematoma, which not only provides a good culture medium for the growth of bacteria, but also increases the risk of infection, and may also oppress surrounding tissues and affect local blood circulation. Blood circulation disorder will affect the nutrient supply of cartilage, lead to cartilage absorption and necrosis, and then affect the surgical effect. In addition, massive blood loss may also cause stress reaction of patients, affect immune system function, and indirectly increase the risk of complications (12, 13). Related research shows that the incidence of postoperative complications increases significantly when the amount of intraoperative bleeding exceeds a certain threshold. The thin skin thickness of nasal dorsum is an important anatomical factor affecting the effect and complications of rhinoplasty. The skin on the back of the nose is too thin to provide sufficient tissue coverage and protection for the filled cartilage particles (14, 15). In the process of postoperative recovery, thinner skin is more susceptible to external forces, leading to the displacement of cartilage particles (16, 17). At the same time, thin skin will reduce blood supply, which is not conducive to wound healing, and increase the risk of complications such as skin redness and ulceration (18, 19).

The nomogram prediction model constructed in this study shows good calibration and fitting degree in both training set and verification set, and the C-index index is 0.857 and 0.848, respectively, which shows that the model has good distinguishing ability and can well distinguish patients with and without complications. The area under ROC curve (AUC) is 0.851 in the training set and 0.855 in the verification set, which indicates that the model has high prediction efficiency. The analysis of decision curve shows that the nomogram model has obvious clinical benefits in predicting postoperative complications within a certain threshold probability range, and can provide valuable reference for clinical decision-making (20, 21). However, this study also has some limitations. This study only carried out internal verification, and did not carry out external verification. This is mainly because external verification needs to collect many case data from other medical institutions, and it faces many difficulties in actual operation, such as the differences in surgical operation norms and patient management modes of different medical institutions, and the difficulty in ensuring the accuracy and completeness of data collection. In addition, this study is a single-center study, and the representativeness of the sample may be limited. Follow-up research should actively carry out multi-center, large-sample research and external verification, to further verify and improve the nomogram prediction model and improve the accuracy and universality of the model (22). Thirdly, while we recorded detailed patient characteristics, the sample size precluded meaningful subgroup analyses that might have identified nuanced risk factors. Future multicenter studies with larger cohorts should incorporate predefined stratification by surgical history, systemic conditions, and technical variables to validate and extend our findings. Such investigations would help establish personalized risk prediction models for broader clinical application. Fourthly, the small severe complication cohort precluded meaningful severity-stratified analyses without compromising statistical power. While this binary approach ensures model robustness, future multicenter studies should prioritize larger samples to enable graded complication analyses (mild/moderate/severe) and refine risk stratification. In addition, all surgical indications in this series were purely aesthetic, with preoperative otolaryngologic examination confirming normal nasal function in all patients. While this provides a homogeneous population for analyzing aesthetic complication factors, the findings may not generalize to functional or reconstructive cases—an important direction for future study.

To address the limitation of lacking external validation, we plan to implement multi-center external validation study in the next 1–2 years. The specific roadmap includes: 1. collaborating with 3–5 tertiary hospitals with rich experience in rhinoplasty to ensure the generalizability of the model across different clinical settings; 2. establishing unified inclusion and exclusion criteria consistent with this study to minimize selection bias; 3. collecting a target sample size of at least 200 cases from collaborating centers, with detailed recording of variables included in the nomogram (age, chronic rhinitis history, surgical duration, intraoperative blood loss, nasal dorsal skin thickness) and postoperative complications; 4. validating the model using consistent statistical metrics (C-index, AUC, calibration curve, Hosmer-Lemeshow test) to evaluate its discrimination and calibration in external populations; 5. adjusting the model parameters if necessary based on external validation results to optimize its adaptability to diverse clinical scenarios. This multi-center external validation aims to confirm the model's generalizability and prepare it for clinical use. By including diverse hospitals (e.g., from northern, eastern, and southern China) with varying patient demographics, surgical practices, and care protocols, we ensure robustness across different settings. Subgroup analyses by institution type and region will further reduce bias, strengthening the model's real-world applicability.

To sum up, this study analyzed the clinical data of 214 patients who underwent closed approach autogenous costal cartilage augmentation rhinoplasty, and determined that older age, history of chronic diseases (chronic rhinitis), long operation time, large amount of blood loss during operation and thin skin thickness on the back of nose were independent risk factors for postoperative complications. The nomogram prediction model based on these risk factors shows good calibration and discrimination in both training set and verification set, and has high prediction efficiency, which can provide a convenient and effective tool for clinicians to predict postoperative complications. This model is helpful for clinicians to make individualized risk assessment of patients before operation, and make reasonable surgical plan and perioperative nursing plan according to the specific situation of patients, to minimize the risk of complications and improve the surgical effect and patient satisfaction. Although there are some limitations in this study, it lays a foundation for the follow-up research. In the future, it is necessary to carry out multi-center and large-sample research and external verification, further improve the nomogram prediction model, make it play a greater role in clinical practice, and bring better treatment results to most patients.

## Data Availability

The raw data supporting the conclusions of this article will be made available by the authors, without undue reservation.
